# Tissue Specific Modulation of cyp2c and cyp3a mRNA Levels and Activities by Diet-Induced Obesity in Mice: The Impact of Type 2 Diabetes on Drug Metabolizing Enzymes in Liver and Extra-Hepatic Tissues

**DOI:** 10.3390/pharmaceutics9040040

**Published:** 2017-09-26

**Authors:** Sarah Maximos, Michel Chamoun, Sophie Gravel, Jacques Turgeon, Veronique Michaud

**Affiliations:** 1 Centre de recherche du Centre hospitalier de l’Université de Montréal (CRCHUM), Montreal, QC H2X 0A9, Canada; sarah.maximos@umontreal.ca (S.M.); sophie.gravel.3@umontreal.ca (S.G.); turgeoja@gmail.com (J.T.); 2 Faculty of Medicine, Université de Montréal, Montreal, QC H3T 1J4, Canada; 3 Faculty of Pharmacy, Université de Montréal, Montreal, QC H3T 1J4, Canada; michel_chamoun94@hotmail.com

**Keywords:** cytochromes P450, drug metabolism, mRNA, diet induced obesity, diabetes

## Abstract

Various diseases such as type 2 diabetes (T2D) may alter drug clearance. The objective of this study was to evaluate the effects of T2D on CYP450 expressions and activities using high-fat diet (HFD) as a model of obesity-dependent diabetes in C57BL6 mice. The cyp450 mRNA expression levels for 15 different isoforms were determined in the liver and extra-hepatic tissues (kidneys, lungs and heart) of HFD-treated animals (*n* = 45). Modulation of cyp450 metabolic activities by HFD was assessed using eight known substrates for specific human ortholog CYP450 isoforms: in vitro incubations were conducted with liver and extra-hepatic microsomes. Expression levels of cyp3a11 and cyp3a25 mRNA were decreased in the liver (>2–14-fold) and kidneys (>2-fold) of HFD groups which correlated with a significant reduction in midazolam metabolism (by 21- and 5-fold in hepatic and kidney microsomes, respectively, *p* < 0.001). HFD was associated with decreased activities of cyp2b and cyp2c subfamilies in all organs tested except in the kidneys (for tolbutamide). Other cyp450 hepatic activities were minimally or not affected by HFD. Taken together, our data suggest that substrate-dependent and tissue-dependent modulation of cyp450 metabolic capacities by early phases of T2D are observed, which could modulate drug disposition and pharmacological effects in various tissues.

## 1. Introduction

Type 2 diabetes (T2D) has become a worldwide public health concern as prevalence of the disease continues to rise [1]. In 2014, the American Diabetes Association reported that 29.1 million Americans, or 9.3% of the population, had T2D [2]. In addition to anti-diabetic drugs, T2D patients commonly require multiple drug therapies to treat a wide range of comorbidities such as hypertension, stroke, dyslipidemia, atherosclerosis and coronary artery disease [3]. Clinical practice reveals that T2D patients show highly variable pharmacokinetics and responses to several drugs used to treat T2D and its related comorbidities [4,5,6,7]. For instance, variable drug dosages and effects are observed for drugs such as clopidogrel, warfarin, cyclosporine and tacrolimus, as well as for anti-hypertensive and cholesterol lowering drugs [8,9,10,11,12,13,14]. Hence, the treatment of co-morbidities in T2D is associated with variable drug response and unexpected toxicities [4,15,16,17].

Currently, information available on the underlying mechanisms responsible for this variability is uncertain. Patients with T2D have a high prevalence of metabolic syndrome (85% vs. 24% in general population), which is associated with a chronic low-grade inflammatory state [18,19,20]. Several reports showed that some inflammatory mediators may modulate expression levels and activities of numerous proteins including some isoenzymes of the cytochrome P450 (CYP450) superfamily [21,22,23,24]. In fact, interleukin-B (IL-1B), interleukin-6 (IL-6) and interferon-γ (INF-γ) have been associated with decreased expressions and activities of CYP450s, especially of CYP3A, in cultured human hepatocytes [25,26,27]. It is also known that pathophysiological changes resulting from obesity affect drug-metabolizing enzyme expressions and activities [28,29,30].

In humans, some studies have reported a decrease in CYP3A4 and an increase in CYP2E1 activities with obesity, while its effects on other isozymes remain uncertain [31,32,33,34]. For instance, Woolsey et al. have reported that CYP3A activity and CYP3A4 mRNA expression were reduced in humans and mice with nonalcoholic fatty liver disease [35]. However, results from animal studies are inconsistent from one study to the other [30,36,37,38,39,40,41,42]. These discrepancies could be explained by the type of diabetes being studied (i.e., T1D vs. T2D), and the strategy used to induce diabetes (i.e., genetically-modified animals vs. chemicals vs. diet). For instance, down-regulation of CYP1A2 and CYP3A1, and up-regulation of CYP3A2 are observed in Goto–Kakizaki rats (genetic model of non-obese T2D) [43]. Conversely, alloxan or streptozocin were associated with an increase expression of hepatic CYP1A2, CYP2B1/2, CYP3A1/23 and CYP2E1 in diabetic rats compared to controls [44,45,46,47,48,49]. No alteration in hepatic levels of cyp1a2 and cyp2e1 has been found in streptozocin-induced diabetic mice [41]. A study from Ghose et al. revealed a decrease of cyp3a activity, but unaffected cyp1a2 and cyp2e1 activities in high-fat diet (HFD) fed mice [38]. Finally, no difference was observed for cyp3a11 expression or activity in db/db mice (a genetic T2D mouse model) [37].

Several organs express various combinations of CYP450s and, thus, different patterns of CYP450 expression in tissues may be a key determinant of variability observed for drug response. Although extra-hepatic CYP450 activity is manifested to a lower magnitude compared to the liver [50], variability in tissue-specific metabolizing CYP450 enzymes may lead to variation in drug effects due to local metabolism in target organs. To date, there is a paucity of information on the influence of pathophysiological conditions such as T2D on the activity and expression of extra-hepatic CYP450s.

In this study, we sought to determine the effects of T2D on the expression and activities of hepatic and extra-hepatic CYP450 enzymes using the high fat-diet (HFD) diet-induced obesity (DIO) C57BL6 mouse as a T2D model [51]. DIO mice were stratified into two groups according to the effect of HFD on their body weight at the end of the treatment period: low-diet responders and high-diet responders. These two groups have been well characterized and correspond to the early diabetes situation observed in obese humans [51]. An extensive phenotyping characterization was conducted in many organs (liver, kidneys, lungs and heart) to assess the tissue-specific modulation of cyp450 expressions and activities. 

## 2. Materials and Methods

### 2.1. Chemicals

Ebastine, hydroxyebastine, carboxyebastine, desalkylebastine, hydroxyebastine-d5, carebastine-d5, desalkylebastine-d5, bufuralol, hydroxybufuralol, hydroxybufuralol-d9, repaglinide, 2-despiperidyl-2-amino repaglinide (M1), 3′-hydroxyrepaglinide (M4), bupropion, hydroxubupropion, hydroxybupropion-d5, hydroxytolbutamide, carboxytolbutamide, 1′-hydroxytolbutaminde-d9, 4′-carboxytolbutamide-d9, 4-hydroxymidazolam, 4-hydroxymidazolam-d5, 6-hydroxychlorzoxazone and 12-hydroxydodecanoic-d20 acid were purchased from Toronto Research Chemicals (Toronto, ON, Canada). Hydroxychlorzoxazone-d2 was obtained from TLC PharmaChem (Mississauga, ON, Canada). Midazolam and 1′-hydroxymidazolam-d4 were purchased from Cerilliant (Round Road, TX, USA). Chlorzoxazone, tolbutamide, dodecanoic acid, β-Nicotinamide-Adenine Dinucleotide Phosphate (NADP), glucose-6-phosphate (G6P), glucose-6-phosphate dehydrogenase (G6PD), dimethyl sulfoxide (DMSO), trishydroxymethylaminomethame (TRIS), and phenylmethanesulfonyl (PMSF) were purchased from Sigma-Aldrich (St. Louis, MO, USA). Ethynediaminetetraacetic acid (EDTA) and dithiothreitol were obtained from Bishop (Burlington, ON, Canada) and from Gibco^®^, Life Technologies Ltd. (Eugene, OR, USA), respectively. All other chemicals used were commercially available and were of analytical grade.

### 2.2. Animals

Five-week-old male C57BL/6 mice (*n* = 45) were purchased from Charles River Laboratories (Montreal, QC, Canada). Animals were housed in a temperature-, light-, and humidity-controlled environment, they were housed 2 per cage and were maintained at an ambient temperature of 21 °C on a 12-h light/dark cycle with free access to water and food ad libitum. The obese diabetic mice were developed according to the experimental protocol described previously [51]. Briefly, one week after their arrival, mice (mean weight 20.0 ± 1.0 g) were fed with a high-fat diet (HFD) (Bio-Serv Diet #F3282, Frenchtown, NJ, USA, 60% fat by energy) or the standard normal diet (ND) (Teklad Global 18% protein diet; Harlan Teklad, Madison, WI, USA, 15% fat by energy) for 8 weeks. Body weight was measured weekly while blood glucose and insulin were determined at Week 8. After 8 weeks of diet, HFD mice were stratified according to their body weight and two groups were formed as follows; low responders to HFD (LDR) (<39.9 g) and high responders to HFD (HDR) (39.9–45 g). Peyot et al. have demonstrated that the LDR are less obese, develop intermediate severity of insulin resistance and have mild impairment in glycemia, while the HDR are more obese, insulin resistant, hyperinsulinemic and hyperglycemic [51]. Animals were sacrificed by cervical dislocation, and organs including heart, lungs, kidneys and liver were quickly excised, washed with cold TRIS 100 mM buffer (pH 7.4) and immersed in liquid nitrogen (−80 °C). Experimental protocols were approved by the institutional committee of animal protection and were carried out in accordance with the Guide for the Care and Use of Experimental Animals of the Canadian Council on Animal Care.

### 2.3. CYP450 mRNA Levels

#### 2.3.1. Isolation of RNA and Preparation of cDNA

For each organ tested (using a pool of 3 mice/organ), about 100 mg of tissue was homogenized in 1 mL of Trizol and incubated for 5 min at room temperature. Chloroform (200 µL) was added, the mixture shaken for 15 s and then, centrifuged at 16,000× *g* for 30 min at 4 °C. The aqueous supernatant (500 µL) was transferred and ethanol 70% was added (1:1 *v*/*v*). RNA was extracted using the Qiagen kit (RNeasy Mini kit; Qiagen Sciences, MD, USA) according to the manufacturer’s recommendations. RNA concentration and quality was assessed by spectrometry. Total RNA (2 µg) from each sample was used for reverse transcription. RNA, random primers (6 µg) and dNTP (25 mM) were preheated for 5 min at 65 °C. Then, 5X-first strand buffer, 80 units of RNAse inhibitor, DTT (0.01 M) and 400 units of Superscript II (Invitrogen, Carlsbad, CA, USA) were added to a final volume of 40 µL. Reverse transcription was carried out for 50 min at 42 °C and stopped by heating to 70 °C for 15 min (final RNA concentration 50 ng/µL). The resulting cDNA was frozen at −80 °C until analyzed.

#### 2.3.2. RT-qPCR Analysis

Real-time quantitative PCR was performed using TaqMan^®^ probe and primer sets from Applied Biosystem (Foster, CA, USA). The assay IDs for selected cyp450s were: cyp2b9 (Mm00657910_m1), cyp2b10 (Mm01972453_s1), cyp2c29 (Mm00725580_s1), cyp2c37 (Mm00833845_m1), cyp2c39 (Mm04207909_g1), cyp2c40 (Mm04204172_mH), cyp2d9 (Mm00651731_m1), cyp2d10 (Mm00731648_m1), cyp2d22 (Mm00530542_m1), cyp2e1 (Mm00491127_m1), cyp2j5 (Mm00487292_m1), cyp2j6 (Mm01268197_m1), cyp3a11 (Mm00731567_m1), cyp3a13 (Mm00484110_m1), cyp3a25 (Mm01209536_m1) and cyp4a10 (Mm01622743_g1). As reference genes, gapdh (Mm99999915_g1) and b2m (Mm00437762_m1) were used as housekeeping genes. cDNA was diluted to 10 ng/reaction, mixed with TaqMan^®^ PCR Master Mix (10 µL) and amplified using cycling conditions as follows: 45 cycles consisting of 10 s at 95 °C and 45 s at 60 °C. Reactions were run in a QuantStudio 6 Flex System (Life Technologies Inc., Burlington, ON, Canada).

The relative quantification of various gene expressions was calculated to the comparative CT method using the formula 2^−ΔCT^ [52,53]. Only CT values ≤ 35 were included in the analyses. Since CT values > 35 were not reliable and considered below the detection level of the assay, a CT value of 35 to 38 was defined not quantifiable (NQ) while a value of CT > 38 as not detectable (ND). For their part, mRNA levels associated with the expression of each isoenzyme under a specific diet condition (ND, LDR, HDR) were determined using a calibrator and the following formula 2^−ΔΔCT^ [52]. The calibrator was prepared at the same mRNA concentration using a pool of RNA obtained for each tissue (Clontech A Takara, Bio Company, Mountain View, CA, USA). Determination of mRNA levels was performed in triplicate for each sample, and three independent experiments were repeated to confirm results.

### 2.4. In Vitro CYP450 Metabolism in Liver and Extra-Hepatic Organs

#### 2.4.1. Preparation of Microsomes

Microsomes from liver, kidneys, lungs and heart were prepared according to our previously described methods with slight modifications [54]. Briefly, tissue (pools of organs from 3 mice) was homogenized in an ice-cold buffer consisting of 50 mM-150 mM-1 mM TRIS-KCL-EDTA buffer (liver and kidneys) or 100 mM-150 mM-1 mM PO_4_-KCL-EDTA buffer (lungs and heart) and both buffers containing protease inhibitors namely, PMFS (0.01 mM) and DTT (0.5 mM). Microsomal subcellular fraction was prepared by centrifugation (10,000 *g* × 20 min, at 4 °C) followed by ultra-centrifugation (100,000 *g* × 90 min, at 4 °C). The microsomal pellets were resuspended in the same buffer (without PMSF and DTT), and frozen at −80 °C until in vitro metabolism experiments were performed. The protein concentration of the microsomes was determined by the Bradford method using bovine serum albumin as the standard.

#### 2.4.2. Effects of the HFD on Hepatic and Extra-Hepatic cyp450 Activities

In order to investigate the effects of the HFD as a representative model of type 2 diabetes on cyp450 activities, in vitro incubations were performed in presence of various microsomes with several probe drugs of CYP450s including bupropion, repaglinide, tolbutamide, bufuralol, chlorzoxazone, ebastine, midazolam and dodecanoic acid, which were used as markers of the functional orthologs of human CYP2B6, CYP2C8, CYP2C9, CYP2D6, CYP2J2, CYP2E1, CYP3A4/5 and CYP4A11, respectively. This study employed cocktails of probe substrates already accepted and validated to investigate the impact of diabetes on specific cyp450 activities. The production of each specific metabolite was quantified from substrate probes and variations of in vitro probes reactions can be inferred to affect all substrates metabolized by the same enzymes.

Assay conditions were previously optimized by standard incubations with probes (buffer, incubation period, protein contents and drug concentrations). All incubations were performed in triplicate. The incubation mixture containing microsomes [5 µL for liver (~20 mg proteins/mL) and 50 µL for kidney, lungs and heart microsomes (~7–14 mg proteins/mL)], NADPH-regenerating system solution (NADP 6.5 mM, G6P 16.5mM, MgCl_2_ 5 mM and 0.2 U G6PD) and 100 mM phosphate buffer PO_4_ (pH 7.4) or 100 mM TRIS buffer (pH 7.4, for tolbutamide and dodecanoic acid) were pre-incubated in a shaking bath for 10 min at 37 °C. Reaction was initiated by the addition of substrates (bupropion, tolbutamide, bufuralol, chlorzoxazone, midazolam, ebastine, dodecanoic acid or repaglinide) to the incubation mixture (total final volume of 500 µL). Bupropion, chlorzoxazone, ebastine and midazolam were incubated together as a cocktail as previously described whereas the other probe substrates were tested separately. The substrate concentrations used span a range from 38–620 μM for bupropion, 2.5–40 μM for bufuralol, 50–800 μM for chlorzoxazone, 0.125–2 μM for ebastine, 1.25–20 μM for dodecanoic acid, 0.25–4 μM for midazolam, 0.85–14 μM for repaglinide and 25–400 μM for tolbutamide (5 different concentrations were used with liver microsomes while one concentration (>2 km) to ensure saturation was selected to investigate cyp450 activities in extra-hepatic microsomes). After 30 min, the reaction was stopped using 1000 µL of ice-cold internal methanol containing isotope-labeled internal standard probe metabolite(s). Reaction mixtures were put on ice for 10 min, and following a centrifugation at 13,000 rpm for 10 min, and then the supernatant was transferred for analysis. For dodecanoic acid metabolite analysis, the solution was evaporated to dryness at 50 °C under a gentle stream of nitrogen, reconstituted with 200 µL of methanol and transferred to an injection vial for analysis.

### 2.5. High Performance Chromatography–Mass Spectrometry Analytical Methods

#### 2.5.1. Chromatographic Conditions for the Metabolites of Bupropion, Midazolam and Ebastine

This analysis was performed on a Thermo Scientific Acclaim RSLC Polar Advantage C16 column (75 mm × 3.0 mm, 3 µm) and Phenomenex Security Guard Cartridge (C12, 4 mm × 2 mm) operating at 50 °C. The mobile phase was a gradient elution consisting of (A) 0.1% formic acid in acetonitrile and (B) 10 mM ammonium formate in water adjusted to pH 3; ratio A:B varied from 25:75 to 60:40 (*v*/*v*), at a flow rate 500 µL/min (a total run time of 10 min). A 10 µL aliquot of the extract was injected into LC-MSMS system.

A Thermo Scientific TSQ Quantiva Triple Quadrupole mass spectrometer (San Jose, CA, USA) was interfaced with a Thermo Scientific Ultimate 3000 XRS UHPLC system (San Jose, CA, USA) using a pneumatic assisted heated electrospray ion source. MS detection was performed in positive ion mode using selected reaction monitoring (SRM). Selection of optimal transitions and collision energy and tube lens voltage conditions for the metabolites and their respective internal standard are listed in Table S1.

#### 2.5.2. Chromatographic Conditions for the Metabolites of Chlorzoxazone, Tolbutamide, Dodecanoic Acid, Bufuralol and Repaglinide

These analyses were carried out on a Phenomenex Luna PFP (2) column (150 mm × 3.0 mm I.D., 3 μm) with a Phenomenex PFP security guard cartridge operating at 40 °C. A mobile phase in isocratic mode was composed of acetonitrile and 0.01% formic acid having a fixed ratio of 40:60 (*v*/*v*) for chlorzoxazone, tolbutamide and dodecanoic acid, while a ratio of 50:50 was used for bufuralol and repaglinide. The flow rate was 0.30 mL/min (total run time 10 min). A 10 µL aliquot of the extract was injected into the LC-MSMS system.

The HPLC system consisted of a Shimadzu Prominence series UFLC pump and auto sampler (Kyoto, Japan). The tandem MS system used was a Thermo TSQ Quantum Ultra (San Jose, CA, USA). The mass spectrometer was interfaced with the HPLC system using a pneumatic assisted heated electrospray ion source. MS detection was performed in negative ion mode for chlorzoxazone, tolbutamide, and dodecanoic acid metabolites, and in positive ion mode for bufuralol and repaglinide metabolites using selected reaction monitoring (SRM). Selection of optimal transitions and collision energy and tube lens voltage conditions for the metabolites and their respective internal standard are shown in Table S2.

### 2.6. Statistical Analysis

Calibration curves were calculated from the equation *y* = a*x* + b, as determined by weighted 1/*x* and 1/*x*^2^ linear regressions of the calibration lines constructed from the peak-area ratios of metabolites to the internal standard (XCalibur software, Thermo Fisher, San Jose, CA, USA). Relative expression levels of CYP450s mRNAs were analyzed by one-way analysis of variance followed by the Dunnett post-hoc test. A difference with *p* < 0.05 was considered statistically significant. Enzyme kinetic parameters were determined by non-linear regression analysis using Michaelis–Menten equation and Lineweaver–Burk double reciprocal plot and data points were expressed as the mean ± S.D., *K*_m_ and *V*_max_ values and the 95% confidence interval for the intrinsic clearance. Data were analyzed using GraphPad Prism 5 (GraphPad Software, La Jolla, CA, USA) and SAS statistical software (Version 9.4 of the SAS System for Windows, Copyright©, SAS Institute Inc., Cary, NC, USA).

## 3. Results

### 3.1. Animal Model

Although no animal model exactly reflects human T2D, some have similar features. Human T2D is a heterogeneous disorder with a complex interplay between genetic, epigenetic and environmental factors. On one hand, diabetic animal models including chemically-induced or surgically-provoked develop hyperglycemia primarily by cytotoxic actions on beta cells rather than through insulin resistance. On the other hand, transgenic/knockout model are more useful to investigate the role of a specific candidate gene unlike heterogeneity as seen in humans. Moreover, the observed diabetes conditions are less stable; chemicals can produce toxic actions and development of digestive problems can be observed which could also affect CYP450 activities. Consequently, a validated nutritionally (high-fat diet; HFD) obese mouse model has been selected to characterize the effects of obesity-induced diabetes on CYP450 activities [51].

Weight, glycemia and insulinemia measured at week 8 were as follow; 29.2 ± 1.1 g, 9.7 ± 0.3 mmol/L and 1.37 ± 0.35 ng/mL in the chow-fed control group (*n* = 12); 36.4 ± 0.9 g, 9.4 ± 0.5 mmol/L and 2.59 ± 0.44 ng/mL for the LDR group (*n* = 12) and 43.1 ± 0.6 g, 10.5 ± 0.4 mmol/L and 6.82 ± 1.32 ng/mL, for the HDR group (*n* = 16), respectively. Weight, glycemia and insulinemia parameters were significantly higher for HFD groups compared to the control group (*p* < 0.05). Five mice were considered as extreme responders (outliers) since their weight was over 45.0 g and were excluded from our analysis.

### 3.2. Effects of HFD on cyp450 mRNA Expression Levels

#### 3.2.1. General Pattern of cyp450 Expression

**Figure 1:**
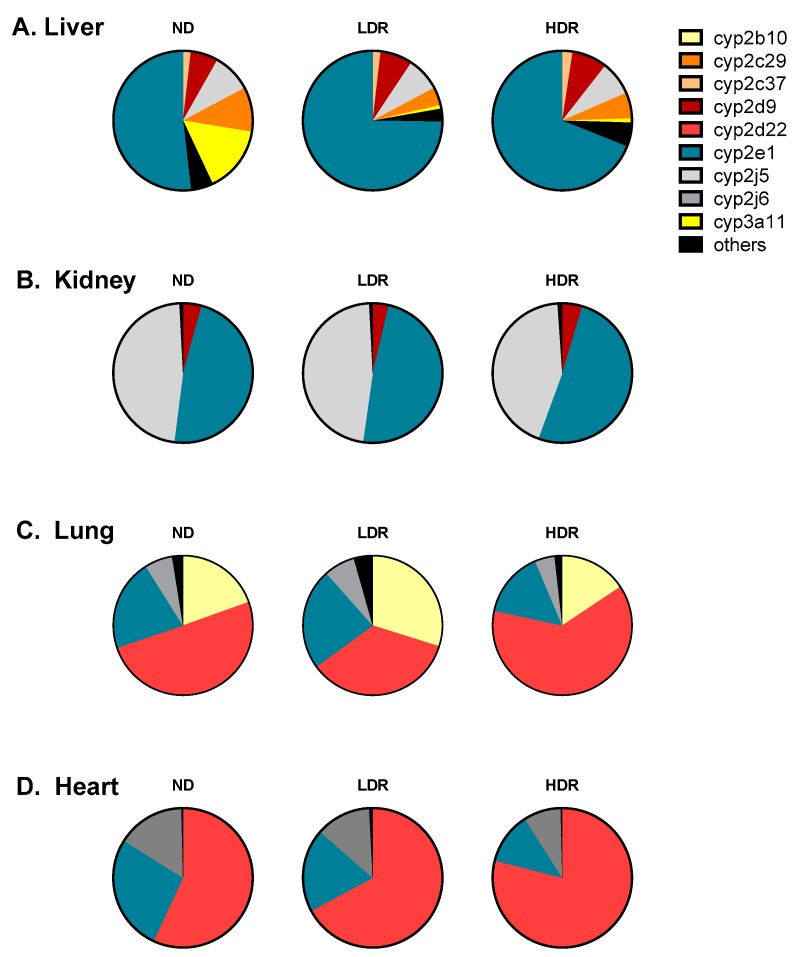
Cyp450 mRNA pie charts. Total mRNA transcripts for each isoenzyme are displayed as expressed in C57BL6 mouse microsomes according to diet group: (**A**) Liver; (**B**) Kidney; (**C**) Lung; and (**D**) Heart. ND, Normal diet; LDR, low-diet responders; and HDR, high-diet responders. Cyp450 mRNA transcript with a relative contribution >1% are illustrated, and “others” have a relative contribution <1%. Others include the following isoforms: Liver, cyp2b10, cyp2b29, cyp2c39, cyp2c40, cyp2d22, cyp2j6, cyp3a13, cyp3a25, and cyp4a10; Kidneys, cyp2b10, cyp2b29, cyp2c29, cyp2c37, cyp2c39, cyp2c40, cyp2d22, cyp2j6, cyp3a11, cyp3a13, cyp3a25, and cyp4a10; and Lungs, cyp2b29, cyp2c29, cyp2c37, cyp2c39, cyp2c40, cyp2d9, cyp2j5, cyp3a11, cyp3a13, cyp3a25, and cyp4a10.

The relative expression of total cyp450 mRNA levels for 15 cyp450 isoforms found in hepatic and extra-hepatic tissues are illustrated for chow-fed control group (ND) and HFD groups in Figure 1 (and Table S3). Major differences were observed in the expression pattern of various cyp450s among tissues. On the other hand, the pattern of cyp450 expression was rather similar among the different diet group: hence, the HFD did not change the pattern of relative cyp450 expression levels in a specific organ. The highest relative levels of mRNAs were cyp2e1 in the liver, cyp2j5/cyp2e1 in kidneys, and cyp2d22 in both heart and lung tissues. The cyp2d, cyp2e and cyp2j subfamilies were expressed in all organs tested (i.e., liver, kidneys, heart and lungs). Moreover, high levels of cyp2b10 (representing approximately 20% of cyp450 mRNA expression) were also observed in the lungs.

#### 3.2.2. Modulation of cyp450 mRNA Expression by HFD

Table 1 presents the relative mRNA transcripts for each cyp450 isoform found in each individual organ under HFD (LRD and HRD) vs. normal chow-diet (ND). Overall, our results showed that HFD altered the profile of mRNA expression in an isoenzyme-specific manner. Moreover, HFD altered the expression profile of cyp450 mRNAs in a tissue-specific fashion. Our major findings were: (1) mRNA levels of cyp3a11 were significantly decreased (approximately 14-fold) in the liver of both HFD groups (LRD and HRD) compared to ND (*p* < 0.001); (2) in contrast, a two-fold increase of hepatic mRNA levels of cyp2b9 (*p* < 0.001), cyp2c39 (*p* < 0.01) and cyp4a10 (*p* < 0.05) were observed in HDR compared to ND; and (3) cyp2b10 (*p* < 0.01) was also increased by two folds in the lungs.

### 3.3. Modulation of cyp450 Hepatic Activities by DIO Mouse as a Model of T2D

#### 3.3.1. Hepatic Activities

**Figure 2:**
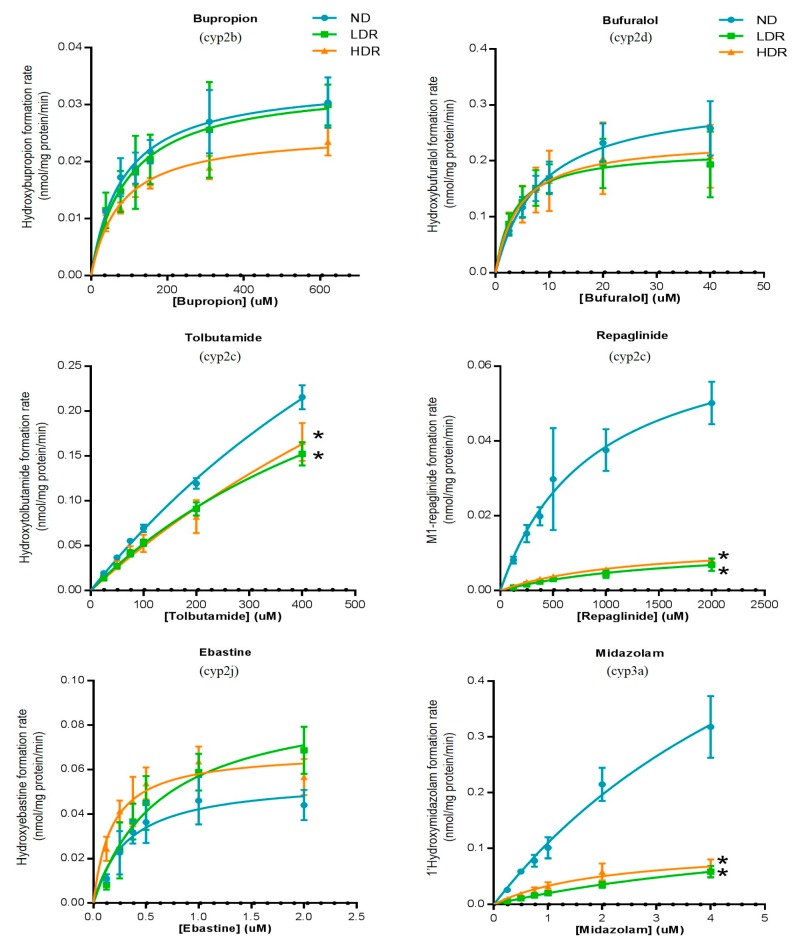

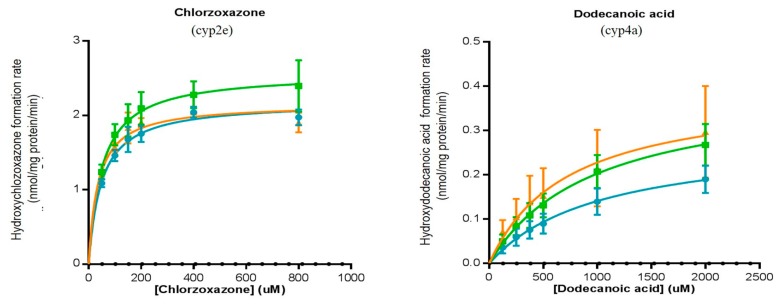
Hepatic cyp450 activities of cyp2b (bupropion), cyp2c (tolbutamide and repaglinide), cyp2d (bufuralol), cyp2e (chlorzoxazone), cyp2j (ebastine), cyp3a (midazolam) and cyp4a (dodecanoic acid) in C57BL6 mice fed a normal diet (ND) or a HFD (LDR, low-diet responders; and HDR, high-diet responders after an eight-week period of treatment). Data were expressed as the mean ± S.D. LDR or HDR vs. ND; * *p* < 0.05. N/F; activity not found, N/A; Not Available (in vitro incubation could not be determined) and dotted line represents the limit of quantification.

As shown in Figure 2 (Table 2), HFD induced variations in hepatic cyp450 activities in an isoform-dependent manner. A significant decrease in midazolam metabolism, a marker of cyp3a subfamily, was observed following HFD treatment; the intrinsic clearance of 1-hydroxymidazolam was reduced in LDR and HDR (23 and 40 µL/min/mg prot) groups compared to ND (107 µL/min/mg prot) (*p* < 0.001) (Table S4). This was mostly explained by a decrease in V_max_; formation of 1-hydroxymidazolam decreased from 0.32 (ND) to 0.06 nmol/mg protein/min (LDR and HDR, *p* < 0.001) (Table 2). HFD treatment was also associated with a diminished hepatic activity of cyp2c subfamilies compared to ND group. The intrinsic clearance of tolbutamide (used as a probe of CYP2C9 in human) was significantly reduced from 0.80 to 0.54 and 0.57 µL/min/mg prot in ND, LDR and HDR groups, respectively (*p* < 0.05) (Table S4). Similarly, HFD affected also the repaglinide hydroxylation (CL_int_ to M1-hydroxyrepaglinide) from 10.6 µL/min/mg prot in ND compared to 1.0–1.4 µL/min/mg prot in HFD groups) (Table S4). Cyp2b activity as measured by bupropion hydroxylation tended to be slightly decreased in HDR group (CL_int_ in HDR vs. ND reduced by 22%). In contrast, no significant effect was observed on the hepatic hydroxylation of bufuralol (cyp2d), dodecanoic acid (cyp4a), chlorzoxazone (cyp2e1) and ebastine (cyp2j) as demonstrated by comparable pharmacokinetics values (Table 2). 

#### 3.3.2. Extra-Hepatic Activities

**Figure 3:**
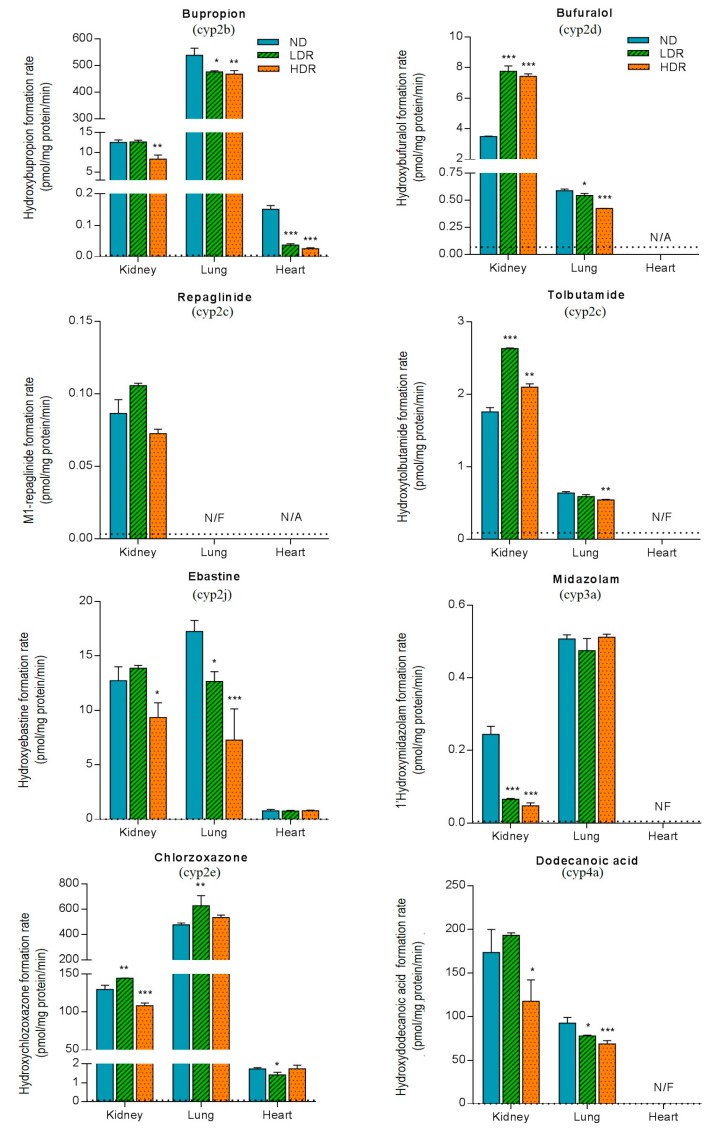
Extrahepatic cyp450 microsomal activities for cyp2b (bupropion), cyp2c (tolbutamide and repaglinide), cyp2d (bufuralol), cyp2e (chlorzoxazone), cyp2j (ebastine), cyp3a (midazolam) and cyp4a (dodecanoic acid) measured in the kidneys, lungs and heart in C57BL6 mice fed a normal diet (ND) or a HFD (LDR, low-diet responders; and HDR, high-diet responders after an eight-week period). Bars and error bars represent the mean ± SD, respectively. LDR or HDR vs. ND; * *p* < 0.05, ** *p* < 0.01, *** *p* < 0.001. N/F; activity not found, N/A; Not Available (in vitro incubation could not be determined) and dotted line represents the limit of quantification.

Figure 3 (Table 3) illustrates the effects of diabetes induced by HFD on the formation rate of cyp450 probe metabolites measured in extra-hepatic tissues. A tissue-dependent modulation of cyp450 activities by HFD was observed. In the kidneys, HFD treatment produced a significant decrease in cyp3a activity; formation rate of 1-hydroxymidazolam was three- and five-times lower in LDR and HDR groups, respectively, compared to ND group (*p* < 0.001) (Table 3(A)). In contrast, the cyp3a activity measured in the lung was not affected by the HFD indicating a tissue-dependent modulation of cyp450 by HFD. No cyp3a activity was detectable in mouse hearts.

Our data showed that the formation of hydroxybupropion was approximately 20–50-times greater in the lungs compared to the liver and kidneys (regardless of the diet groups, *p* < 0.0001) (Table 3(B)). Renal and lung microsomes displayed a slight decrease in the hydroxylation of bupropion in HDR group vs. control diet group (*p* < 0.01) (Figure 3). Although the magnitude of activity was low, a similar observation was made for the hydroxylation of bupropion in the heart (0.150 vs. 0.037 and 0.025 pmol/mg protein/min in ND vs. LDR and HDR, respectively, *p* < 0.001) (Table 3(C)). In addition, ebastine (cyp2j) and dodecanoic acid (cyp4a) metabolisms were reduced by HFD, particularly in HDR, in renal and lung tissues. Ebastine hydroxylation in heart microsomes was not affected by HFD, whereas cyp4a activity could not be detected. In contrast, our results showed that DIO mouse were associated with an increase in tolbutamide (~20–50%) and bufuralol (~90–110%) metabolic activities in kidneys, while in lung microsomes, the hydroxylation of tolbutamide and bufuralol tended to decrease (*p* < 0.01 and *p* < 0.001, respectively, in HDR group). Overall, cyp450 activities tended to be reduced in HFD group with greater effects being observed in HDR.

## 4. Discussion

This study demonstrated that expression and activities of major cyp450s involved in the metabolism of drugs were modulated in DIO C57BL6 mice used as a model of T2D (Table S5). First, we demonstrated that cyp3a expression and activities were decreased by HFD. Second, cyp2c activities were reduced in all organs tested (except for tolbutamide in the kidneys). Finally, cyp2b activity, largely expressed in the lungs, was also decreased by HFD.

C57BL/6 mice were divided into three groups: ND, LDR and HDR. LDR were less obese and developed intermediate severity of insulin resistance, while HDR were more obese and developed severe insulin resistance. The extent of changes in cyp450 activities by HFD tended to be gradually decreased in LDR compared to HDR and some effects being only observed in HDR group. This observation suggests that modulation of some cyp450 activities happens at early stage of pre-diabetes and similar pathways of regulation involved in pre-diabetes development can also intervene on cyp450 activities in an isoform-dependent manner.

Obesity and diabetes have been shown to alter the expression and activity of hepatic CYP450s [14,28,29,35,36,37,38,40,41,42,55,56,57]. Clinical studies and animal experiments report mostly on hepatic CYP3A since it is the most important isoenzyme involved in the metabolism of prescribed drugs [58,59]. In humans, a significant decrease in CYP3A activity has been reported in diabetic human liver microsomes compared to healthy subjects [56], while a significant increase in CYP2E1 activity has been reported in obese T2D human liver microsomes [33]. In our study, we observed a significant decrease in hepatic cyp3a mRNA expression levels in HFD groups compared to normal diet group. Similarly, decreased levels of cyp3a11 mRNA expression have been reported in DIO mice [38,59,60]. Preliminary results from our clinical study using oral CYP450 probe cocktail demonstrate that oral clearance of midazolam was significantly reduced in subjects with T2D compared to subjects without T2D [61]. Our results on RNA transcripts are also consistent with phenotypic findings showing that cyp3a activity determined via hydroxylation of midazolam was significantly reduced in the two HFD groups compared to control group. In agreement with our finding, CYP3A activity has been reported to be also decreased in Zucker diabetic fatty (ZDF) rats using midazolam and testosterone as probes [40].

There are no or very limited data pertaining to the effects of T2D on cyp2c and cyp2b families. We observed that cyp2c and cyp2b subfamilies mRNA expression levels were not changed in the liver by HFD compared to normal group, except for cyp2b9 which was increased in HFD groups. Our results are in agreement with those reported by Yoshinari et al. and Guo et al. who demonstrated unchanged relative mRNA levels of cyp2c and cyp2b subfamilies in the liver [59,60]. In contrast, it has been reported a decrease of cyp2b10 mRNA levels in CD1 mice fed with HFD [38]. However, the relative hepatic levels of cyp2b10 mRNA measured in our study were very low or below the limit of quantification yielding a comparison analysis unreliable. In addition, our results showed that hepatic cyp2c metabolic activities, using repaglinide and tolbutamide as markers, were significantly decreased in HFD groups. Kim et al. reported that CYP2C catalytic activity determined with diclofenac was decreased in chemically induced diabetes in rats [62]. This finding, in agreement with our study, suggest that CYP2C activities are impaired, thereby lower metabolic clearance can be anticipated for drugs metabolized by CYP2Cs, particularly CYP2C8 or CYP2C9, under conditions associated with pre-diabetes or diabetes. 

Our results indicate that cyp2e1 mRNA expression levels and activity remained unchanged and comparable among all groups (HFD and ND). In the same way, expression levels of cyp2e1 mRNA were reported to be unchanged in DIO mice [38,59]. In addition, activity of cyp2e1 was also unaffected in DIO and db/db mice [37,38], but increased activity was shown in ZDF [42]. No alteration in hepatic levels of cyp2e1 was found in streptozocin-induced diabetic mice [41]. These differences in cyp2e1 activity modulation could be function of the animal model of diabetes used and the stage of the disease. 

Our data demonstrated a tissue-specific modulation of cyp2b activities by HFD. Indeed, no significant difference was observed for the hydroxylation of bupropion in liver microsomes. However, bupropion hydroxylation was significantly decreased in HFD groups, particularly in the lungs, the heart and kidneys (HDR group only).

To our knowledge, no study has been conducted to assess the impact of HFD induced obesity as a model of early diabetes stage in humans on CYP450s expression or activity in extrahepatic tissues. Although the magnitude of metabolic capacity and significance in total body clearance of extrahepatic CYP450s metabolism are much lower in comparison to hepatic CYP450s, extrahepatic CYP450s metabolism may affect the local exposure to xenobiotics and thus, influence their pharmacological and toxicological effects. For instance, renal cyp450 metabolites of arachidonic acid, 20-HETE and EET, play an important role in the control of blood pressure and the development of acute kidney injury [63]. In fact, arachidonic acid is metabolized by CYP4A and CYP4F families to 20-HETE (vasoconstrictor) and by CYP2C and CYP2J families to EETs (vasodilator). Our results showed a significant decrease in renal activity of cyp4a and cyp2j in the HDR group using dodecanoic acid and ebastine hydroxylation as marker, respectively. This finding indicates that diabetes could influence homeostasis by affecting local biotransformation of endogenous compounds. In high fat diet induced hypertension rats, CYP2C and CYP4A activities were found to be decreased [64]. The discrepancy observed for CYP2C activity can be explained by using different substrate markers (arachidonic acid being not specific for CYP2C but, also a CYP2J substrate).

Little is known about the effects of obesity and diabetes on CYP450 catalytic activities in the lungs, in both humans and animals. Our data showed extensive metabolic activities in lung microsomes for midazolam, bupropion, ebastine and chlorzoxazone corresponding to cyp3a, cyp2b, cyp2j and cyp2e1, respectively. In lung microsomes, HFD was associated with modulation of cyp450 catalytic activity for certain isoforms independently of patterns observed in the liver or in the kidneys. These findings support the concept that CYP450s expressed in the lungs may contribute to drug metabolism for drugs administered intravenously or locally as well as contribute to the first pass metabolism.

In conclusion, the major finding of this study was that HFD affects CYP450 expression and activities in an isoform- and tissue-dependent fashion. Our results clearly indicate that cyp3a and cyp2c metabolic activities were reduced in DIO-T2D mice (Table S5). In humans, these two CYP450 isoenzymes are involved in the metabolism of 80% of medications prescribed in clinical settings. We speculate that modulation of hepatic CYP450s by T2D diabetes may alter drug pharmacokinetics leading to intersubject variability in drug response. In addition, modulation in CYP450s expressed in extra-hepatic organs can cause variations in tissue concentrations of drugs or endogenous compounds leading to impaired pharmacological action of drugs as well as disruption of homeostasis. Therefore, variation in hepatic and extra-hepatic CYP450s makes patients with pre-diabetes and obesity more prone to adverse drug effect, toxicity or inefficacy (e.g., for prodrugs).

## Figures and Tables

**Table 1:** Relative mRNA transcripts for each cyp isoform in C57B/6 mice microsomes according to diet group.

Tissue	Diet	cyp2b9	cyp2b10	cyp2c29	cyp2c37	cyp2c39	cyp2c40	cyp2d9	cyp2d22	cyp2e1	cyp2j5	cyp2j6	cyp3a11	cyp3a13	cyp3a25	cyp4a10
Liver	ND	0.01	0.02	0.49	1.31	0.01	0.44	0.55	0.53	0.56	0.98	1.08	0.58	2.40	0.44	1.25
	LDR	4.89 *	NQ	0.25 *	1.77	0.01	0.20	0.81	0.59	1.02	1.05	0.88	0.04 ^ᵵ^	3.07	0.17	1.22
	HDR	8.51 ^ᵵ^	0.03	0.30	2.15	0.02 **	0.37	0.71	1.03	0.79	0.90	1.09	0.04 ^ᵵ^	2.96	0.21	3.72
Kidney	ND	ND	0.54	2.35	ND	ND	8.62	1.65	1.32	1.28	4.89	4.56	1.41	1.37	2.00	1.42
	LDR	ND	0.46	3.08	ND	ND	3.08	1.35	1.38	1.29	4.68	4.62	0.57	2.12	1.79	1.13
	HDR	ND	0.42	1.59	ND	ND	5.92	1.65	1.50	1.29	4.18	4.03	0.72	2.41	0.95	2.43
Heart	ND	ND	1.21	0.26	ND	ND	ND	0.05	3.24	0.96	0.09	2.19	ND	NQ	ND	1.09
	LDR	ND	2.16	0.49	ND	ND	ND	0.07	3.40	0.62	0.19	1.85	ND	NQ	ND	2.15
	HDR	ND	1.68	0.30	ND	ND	ND	0.07	5.03	0.51 *	0.19	1.67	ND	NQ	NQ	1.47
Lung	ND	ND	0.73	1.11	ND	ND	ND	0.07	2.06	0.21	0.13	1.20	ND	3.26	0.06	3.28
	LDR	ND	1.66 ^ᵵ^	2.96	ND	ND	ND	0.08	2.05	0.33 **	0.47	1.88	ND	8.15 **	0.07	9.90 *
	HDR	ND	1.17 **	1.00	ND	ND	ND	ND	5.76	0.32 *	0.83	1.69	ND	5.39	0.11	3.43

Results are expressed as mean N-fold differences in cyp gene relative to the average expression of housekeeping genes and a calibrator (2^−ΔΔCt sample^). ND, Normal diet; LDR, low-diet responders; and HDR, high-diet responders. NQ = Not Quantifiable (35 < Ct < 38), ND = Not Detectable (Ct > 38). Each experiment was performed three times and in triplicates. One-way ANOVA was performed with Dunnett post-hoc test. LDR or HDR versus ND; * *p* < 0.05, ** *p* < 0.01, ^ᵵ^ *p* < 0.001.

**Table 2:** Hepatic microsome activities for cyp2b (bupropion), cyp2c (tolbutamide and repaglinide), cyp2d (bufuralol), cyp2e (chlorzoxazone), cyp2j (ebastine), cyp3a (midazolam) and cyp4a (dodecanoic acid) in C57BL6 mice fed a normal diet (ND) or a HFD (LDR, low-diet responders; and HDR, high-diet responders after an eight-week period).

Liver	ND	LDR	HDR
	(nmol/mg protein/min)		
Bupropion **→** Hydroxybupropion	0.030 ± 0.004	0.030 ± 0.004	0.023 ± 0.002 **
Tolbutamide **→** Hydroxytolbutamide	0.22 ± 0.01	0.15 ± 0.01 ^ᵵ^	0.17 ± 0.02 ^ᵵ^
Repaglinide **→** M1-repaglinide	0.050 ± 0.006	0.007 ± 0.002 ^ᵵ^	0.0081 ± 0.0003 ^ᵵ^
Repaglinide **→** Hydroxyrepaglinide	0.0020 ± 0.0001	0.0010 ± 0.0002 ^ᵵ^	0.0013 ± 0.0004 **
Bufuralol **→** Hydroxybufuralol	0.26 ± 0.05	0.19 ± 0.06	0.21 ± 0.06
Chlorzoxazone **→** Hydroxychlorzoxazone	2.0 ± 0.1	2.4 ± 0.3 **	1.9 ± 0.1
Ebastine **→** Hydroxyebastine and carebastine	0.044 ± 0.007	0.07 ± 0.01 ^ᵵ^	0.057 ± 0.008 *
Midazolam **→** 1′-hydroxymidazolam	0.32 ± 0.06	0.06 ± 0.01 ^ᵵ^	0.06 ± 0.01 ^ᵵ^
Dodecanoic acid **→** 12-hydroxydecanoic acid	0.19 ± 0.03	0.27 ± 0.05	0.3 ± 0.1 *

Cyp450 activities are reported as the rate of metabolite formation in nmol/mg protein/min ± SD (* *p* < 0.05, ** *p* < 0.01, ^ᵵ^ *p* < 0.001 compared to ND).

**Table 3:** Extra-hepatic microsome activities for cyp2b (bupropion), cyp2c (tolbutamide and repaglinide), cyp2d (bufuralol), cyp2e (chlorzoxazone), cyp2j (ebastine), cyp3a (midazolam) and cyp4a (dodecanoic acid) in C57BL6 mice fed a normal diet (ND) or a HFD (LDR, low-diet responders; and HDR, high-diet responders after an eight-week period): (**A**) renal microsomes; (**B**) lung microsomes; and (**C**) heart microsomes.

**(A)**			
**Kidney**	**ND**	**LDR**	**HDR**
	**(pmol/mg protein/min)**		
Bupropion → Hydroxybupropion	12.5 ± 0.6	12.6 ± 0.4	8 ± 1 **
Tolbutamide → Hydroxytolbutamide	1.76 ± 0.06	2.63 ± 0.01 ^ᵵ^	2.10 ± 0.04 **
Repaglinide → M1-repaglinide	0.09 ± 0.01	0.105 ± 0.002	0.073 ± 0.003
Bufuralol → Hydroxybufuralol	3.50 ± 0.03	7.7 ± 0.4 ^ᵵ^	7.4 ± 0.2 ^ᵵ^
Chlorzoxazone → Hydroxychlorzoxazone	130 ± 6	144.6 ± 0.2 **	108 ± 3 **
Ebastine → Hydroxyebastine and carebastine	13 ± 1	13.9 ± 0.3	9 ± 1 *
Midazolam → 1′-hydroxymidazolam	0.24 ± 0.02	0.065 ± 0.002 ^ᵵ^	0.047 ± 0.008 ^ᵵ^
Dodecanoic acid → 12-hydroxydecanoic acid	173 ± 27	193 ± 3	118 ± 24 *
**(B)**			
**Lung**	**ND**	**LDR**	**HDR**
	**(pmol/mg protein/min)**		
Bupropion → Hydroxybupropion	538 ± 26	476 ± 4 *	467 ± 14 **
Tolbutamide → Hydroxytolbutamide	0.64 ± 0.02	0.59 ± 0.03	0.54 ± 0.01 **
Repaglinide → M1-repaglinide	N/F	N/F	N/F
Bufuralol → Hydroxybufuralol	0.59 ± 0.02	0.55 ± 0.02 *	0.425 ± 0.001 ^ᵵ^
Chlorzoxazone → Hydroxychlorzoxazone	477 ± 16	629 ± 79 **	535 ± 19
Ebastine → Hydroxyebastine and carebastine	17 ± 1	12.6 ± 0.9 *	7 ± 3 ^ᵵ^
Midazolam → 1′-hydroxymidazolam	0.51 ± 0.01	0.47 ± 0.04	0.51 ± 0.01
Dodecanoic acid → 12-hydroxydecanoic acid	93 ± 6	78 ± 1 *	68 ± 4 ^ᵵ^
**(C)**			
**Heart**	**ND**	**LDR**	**HDR**
	**(pmol/mg protein/min)**		
Bupropion → Hydroxybupropion	0.15 ± 0.01	0.037 ± 0.004 ^ᵵ^	0.025 ± 0.003 ^ᵵ^
Tolbutamide → Hydroxytolbutamide	N/F	N/F	N/F
Repaglinide → M1-repaglinide	N/A	N/A	N/A
Bufuralol → Hydroxybufuralol	N/A	N/A	N/A
Chlorzoxazone → Hydroxychlorzoxazone	1.7 ± 0.1	1.2 ± 0.1 *	1.7 ± 0.2
Ebastine → Hydroxyebastine and carebastine	0.8 ± 0.1	0.76 ± 0.06	0.78 ± 0.06
Midazolam → 1′-hydroxymidazolam	N/F	N/F	N/F
Dodecanoic acid → 12-hydroxydecanoic acid	N/F	N/F	N/F

Cyp450 activities determined at 3 km are reported as the rate of metabolite formation in pmol/mg prot/min ± SD (* *p* < 0.05, ** *p* < 0.01 and ^ᵵ^ *p* < 0.001 compared to ND). N/F, activity not found; N/A, Not Available (in vitro incubation could not be determined).

## Supplementary Materials

Tables S1–S5 are available online at www.mdpi.com/1999-4923/9/4/40/s1.

## Abbreviations

DIO	diet-induced obesity
DMSO	dimethyl sulfoxide
G6P	glucose-6-phosphate
G6PD	glucose-6-phosphate dehydrogenase
HDR	high-diet responders
HFD	high-fat diet
IFN-γ	interferon-γ
IL-1β	interleukin-1β
IL-6	interleukin-6
LC-MS/MS	liquid chromatography–tandem mass spectrometry
LDR	low-diet responders
NADP	Nicotinamide-Adenine Dinucleotide Phosphate
P450	cytochrome P450
PMSF	phenylmethanesulfonyl
RT-qPCR	real time quantitative polymerase chain reaction
T2D	type 2 diabetes
VHDR	very high-diet responders
