# Supplementary material for: Tissue Specific Modulation of cyp2c and cyp3a mRNA Levels and Activities by Diet-Induced Obesity in Mice: The Impact of Type 2 Diabetes on Drug Metabolizing Enzymes in Liver and Extra-Hepatic Tissues
Source: Pharmaceutics. 2017 Sep 26;9(4):40. doi: 10.3390/pharmaceutics9040040 (PMC5750646; doi:10.3390/pharmaceutics9040040)
Supplement: Supplementary file 1 [file pharmaceutics-09-00040-s001.pdf]

Tissue Specific Modulation of cyp2c and cyp3a mRNA Levels and Activities by Diet-Induced Obesity in Mice: The Impact of Type 2 Diabetes on Drug Metabolizing Enzymes in Liver and Extra-Hepatic Tissues

Sarah Maximos [1,2], Michel Chamoun [3], Sophie Gravel [1,3], Jacques Turgeon [1,2,3] and Veronique Michaud [1,2,3,*]

[1] Centre de recherche du Centre hospitalier de l'Université de Montréal (CRCHUM), Montreal, QC H2X 0A9, Canada; sarah.maximos@umontreal.ca (S.M.); sophie.gravel.3@umontreal.ca (S.G.); turgeoja@gmail.com (J.T.)
[2] Faculty of Medicine, Université de Montréal, Montreal, QC H3T 1J4, Canada
[3] Faculty of Pharmacy, Université de Montréal, Montreal, QC H3T 1J4, Canada; michel_chamoun94@hotmail.com
* Correspondence: v.michaud@umontreal.ca; Tel: +1-514-890-8000 ext. 158

Table S1. Selected reaction monitoring for bupropion, midazolam and ebastine.

Substrate	Metabolite	SRM (m/z)	Tube Lens (V)	Collision Energy (V)
Bupropion	Hydroxybupropion	256 → 139	91	28
	Hydroxybupropion-d_6	262 → 139	91	28
Midazolam	1′-hydroxymidazolam	342 → 168	98	36
	1′-hydroxymidazolam-d_4	346 → 168	98	36
	4′-hydroxymidazolam	342 → 234	98	22
	4′-hydroxymidazolam-d_5	347 → 235	98	22
Ebastine	Hydroxyebastine	486 → 167	100	31
	Hydroxyebastine-d_5	491 → 172	100	31
	Carebastine	500 → 167	100	29
	Carebastine-d_5	505 → 172	100	29
	Desalkyl-ebastine	268 → 167	91	20
	Desalkyl-ebastine-d_5	273 → 172	91	20

Table S2. Selected reaction monitoring for chlorzoxazone, tolbutamide, dodecanoic acid, bufuralol and repaglinide.

Substrate	Metabolite	SRM (m/z)	Tube Lens (V)	Collision Energy (V)
Chlorzoxazone	Hydroxychlorzoxazone	184 → 120	−65	20
	Hydroxychlorzoxazone-$^{15}Nd_2$	187 → 123	−65	20
Tolbutamide	Hydroxytolbutamide	285 → 186	−50	19
	Hydroxytolbutamide-d_9	294 → 186	−50	19
	Carboxytolbutamide	299 → 200	−65	19
	Carboxytolbutamide-d_9	308 → 200	−65	19
Dodecanoic acid	12-Hydroxydodecanoic acid	215 → 169	−62	21
	12-Hydroxydodecanoic acid-d_{20}	235 → 188	−62	21
Bufuralol	Hydroxybufuralol	278 → 186	77	19
	Hydroxybufuralol-d_9	287 → 187	77	19
Repaglinide	M1-repaglinide	385 → 106	79	33
	Hydroxyrepaglinide	469 → 246	95	25

	Desalkyl-ebastine-d_5	273 → 172	91	20

Table S3. Total mRNA transcripts for each cyp isoform in C57BL6 mice microsomes according to diet group.

	**Liver**			**Kidney**			**Heart**			**Lung**		
	**ND**	**LDR**	**HDR**	**ND**	**LDR**	**HDR**	**ND**	**LDR**	**HDR**	**ND**	**LDR**	**HDR**
cyp2b9	2.85E-05	2.03E-02	3.79E-02	ND	ND	ND	ND	ND	ND	ND	ND	ND
cyp 2b10	6.90E-06	NQ	7.89E-06	7.04E-05	6.32E-05	4.19E-05	1.10E-05	1.57E-05	1.22E-05	8.52E-03	2.55E-02	1.93E-02
cyp 2c29	4.01E-01	1.93E-01	2.43E-01	1.80E-04	2.39E-04	1.21E-04	3.37E-05	7.33E-05	3.70E-05	5.49E-04	1.99E-03	7.19E-04
cyp 2c37	7.08E-02	8.36E-02	9.73E-02	ND	ND	ND	ND	ND	ND	ND	ND	ND
cyp 2c39	2.36E-04	3.35E-04	6.23E-04	ND	ND	ND	ND	ND	ND	ND	ND	ND
cyp 2c40	7.52E-02	3.08E-02	5.12E-02	3.30E-04	1.22E-04	2.21E-04	ND	ND	ND	ND	ND	ND
cyp 2d9	2.48E-01	3.43E-01	3.30E-01	2.28E-01	1.90E-01	2.27E-01	4.38E-06	7.19E-06	6.82E-06	3.85E-05	5.12E-05	ND
cyp 2d22	4.21E-02	4.22E-02	6.62E-02	1.03E-02	1.05E-02	1.16E-02	1.07E-02	1.14E-02	1.92E-02	2.19E-02	3.00E-02	7.73E-02
cyp 2e1	2.01E+00	3.41E+00	2.79E+00	2.51E+00	2.51E+00	2.49E+00	5.04E-03	3.29E-03	2.91E-03	9.27E-03	2.01E-02	1.88E-02
cyp 2j5	3.51E-01	3.58E-01	3.22E-01	2.48E+00	2.42E+00	2.12E+00	1.13E-05	2.11E-05	2.28E-05	5.81E-05	2.20E-04	5.58E-04
cyp 2j6	7.38E-03	5.58E-03	6.93E-03	1.44E-02	1.53E-02	1.32E-02	2.95E-03	2.17E-03	2.09E-03	2.81E-03	6.14E-03	5.67E-03
cyp3a11	6.01E-01	3.79E-02	3.88E-02	2.97E-05	1.20E-05	1.49E-05	ND	ND	ND	ND	ND	ND
cyp 3a13	3.10E-03	3.30E-03	3.15E-03	2.89E-05	4.54E-05	5.80E-05	NQ	NQ	NQ	3.51E-04	1.12E-03	7.57E-04
cyp 3a25	5.84E-02	2.11E-02	2.75E-02	1.22E-05	1.33E-05	5.42E-06	ND	ND	NQ	1.53E-05	2.15E-05	5.20E-05
cyp 4a10	1.30E-02	1.12E-02	3.25E-02	1.88E-02	1.59E-02	2.89E-02	8.49E-06	1.90E-05	1.08E-05	8.43E-05	3.35E-04	1.12E-04

Results are expressed as mean N-fold differences in cyp gene relative to the average expression (using $2^{-\Delta Ct\ sample}$). NQ = Not Quantifiable (35 < Ct < 38), ND = Not Detectable (Ct > 38).

Table S4. The estimated intrinsic clearance values for each substrate-probe in liver microsomes from mice fed a normal diet (ND) or a HFD (LDR; low-diet responders and HDR; high-diet responders after an eight-week period).

	**Intrinsic Clearance (uL/min/mg Protein)**	**95% Confidence Interval**
	Bupropion → Hydroxbuproption	
ND	0.44	[0.40, 0.49]
LDR	0.42	[0.36, 0.51]
HDR	0.37	[0.32, 0.42]
	Tolbutamide → hydroxytolbutamide	
ND	0.80	[0.78, 0.83]
LDR	0.54	[0.48, 0.62]
HDR	0.57	[0.53, 0.63]
	Repaglinide → M1 repaglinide	
ND	10.6	[10.0, 11.2]
LDR	1.0	[0.9, 1.1]
HDR	1.4	[1.4, 1.5]
	Repaglinide → Hydroxyrepaglinide	
ND	0.20	[0.19, 0.21]
LDR	0.10	[0.09, 0.12]
HDR	0.13	[0.13, 0.14]
	Bufuralol → hydroxybufuralol	
ND	39	[37, 41]
LDR	56	[50, 63]

HDR	53	[48, 58]
Chlorzoxazone → Hydroxychlorzoxazone		
ND	43	[39, 48]
LDR	48	[44, 53]
HDR	51	[45, 58]
Ebastine → Hydroxyebastine		
ND	102	[77, 148]
LDR	65	[57, 76]
HDR	285	[245, 341]
Midazolam → 1′-hydroxymidazolam		
ND	107	[102, 11]
LDR	23	[22, 25]
HDR	41	[37, 46]
Dodecanoic acid → Hydroxydodecanoic acid		
ND	30	[26, 35]
LDR	42	[38, 48]
HDR	64	[56, 74]

Table S5. The HFD-induced change on cyp450 activities are summarized for the major drug metabolism enzymes in hepatic and extra-hepatic tissues. Results are expressed as the percentage of change compared to control mouse group (↑ or ↓ %, and—indicating no significant variation). * $p < 0.05$, ** $p < 0.01$, ǂ $p < 0.001$ compared to ND.

Probes	HFD Groups	Liver (%)	Kidney (%)	Lung (%)	Heart (%)
Midazolam	LDR	81% ↓ ǂ	72% ↓ ǂ	-	N/F
(Cyp3a)	HDR	81% ↓ ǂ	80% ↓ ǂ	-	
Tolbutamide	LDR	32% ↓ ǂ	49% ↑ ǂ	-	N/F
(Cyp2c)	HDR	23% ↓ ǂ	19% ↑ **	15% ↓ **	
Repaglinide	LDR	86% ↓ ǂ	-	N/F	N/A
(Cyp2c)	HDR	84% ↓ ǂ	-		
Bupropion	LDR	-	-	12% ↓ *	75% ↓ ǂ
(Cyp2b)	HDR	23% ↓ **	36% ↓ **	13% ↓ **	83% ↓ ǂ
Bufuralol	LDR	-	120% ↑ ǂ	7% ↓*	N/A
(Cyp2d)	HDR	-	111% ↑ ǂ	28% ↓ ǂ	
Ebastine	LDR	59% ↑ ǂ	-	26% ↓*	-
(Cyp2j)	HDR	30% ↑ ǂ	31% ↓ *	59% ↓ǂ	-
Chlorzoxazone	LDR	20% ↑ **	11% ↑ **	32% ↑ **	29% ↓ *
(Cyp2e1)	HDR	-	17% ↓ **	-	-
Dodecanoic acid	LDR	-	-	16% ↓ *	N/F
(Cyp4a)	HDR	-	32% ↓ *	27% ↓ ǂ	

N/A; data not available. N/F; activity not found.
